# Impulse control disorders in very elderly patients with restless legs syndrome

**DOI:** 10.1016/j.prdoa.2019.08.002

**Published:** 2019-08-19

**Authors:** Xiao-Ying Zhu, Yun-Cheng Wu

**Affiliations:** Department of Neurology, Shanghai General Hospital, Shanghai Jiao Tong University School of Medicine, Shanghai 200080, PR China

## 1 Introduction

Impulse control disorders (ICDs) have been widely studied in Parkinson's disease (PD). Dopaminergic pharmacotherapy rather than the PD process is considered the predominant cause of ICDs in PD, although numerous risk factors have been identified correlated with ICDs in PD [1]. Central dopaminergic dysfunction is one of the pivotal roles in restless legs syndrome (RLS) [2], and dopaminergic agonists are frequently used in RLS management [3]. In the past decade, there have also been more and more cases and studies describing ICDs in RLS as complications of dopaminergic medications [[4], [5], [6]]. However, data on ICDs in RLS patients older than 80 are lacking. Here, we describe two cases of ICDs in very elderly patients with RLS after taking dopaminergic drugs. We also discuss issues related to ICDs in elderly RLS patients.

## 2 Case report

### 2.1 Case1

A 92-year-old male complained of leg discomfort for 8 years. He started experiencing unbearable left-side-dominant bilateral leg restlessness when he was 84 years old, which appeared during the night when he was going to sleep and was relieved by getting up and walking. Sometimes he needed a hammer to strike against his legs to seek relief. The leg discomfort was mainly located in the popliteal fossa and back of the lower legs. The patient came to our movement disorder clinic in August 2016 because his symptoms had worsened for several months. Neurological examination, blood tests and brain MRI were all normal. No family history of RLS or other movement disorders was identified. The patient was diagnosed with RLS according to the International Restless Legs Syndrome Study Group (IRLSSG) criteria [7]. Pregabalin was prescribed for therapy but soon withdrawn because of somnolence. Then he received 300 mg oral gabapentin per night 1 h before going to bed, but the medication didn't work. In September 2016, 0.125 mg pramipexole was prescribed nightly for treatment. He felt his leg discomfort was partially alleviated; however, the symptoms fluctuated. Therefore, the dosage of pramipexole was increased to 0.25 mg/d in January 2017. In April 2017, he visited our clinic for an unmentionable complaint of uncontrollable sexual impulses. Since his wife died 4 years ago, he felt it bothered him greatly. On evaluation, his International Restless Legs Syndrome Rating Scale (IRLSRS) score was 27, and the leg restlessness symptoms still troubled him. He was considered as having hypersexuality according to the Questionnaire for Impulsive-Compulsive Disorders in PD (QUIP, kindly provided by Prof. Daniel Weintraub from the University of Pennsylvania). Pramipexole was discontinued and switched to Madopar 125 mg in addition to gabapentin 300 mg. However, he still complained of the torturous increased sexual desire. After discontinuation of Madopar, his compulsive sexual addiction gradually disappeared. During subsequent follow-up visits, the patient didn't take any medication and described no more sexual hyperesthesia, until in late June 2019 he again suffered from two episodes of uncontrollable transient sexual impulse, which soon disappeared after applying a local cold compress. He still had fluctuations of bearable RLS symptoms.

### 2.2 Case 2

An 81-year-old woman had bilateral leg restlessness during the night for 60 years. She came to our movement disorder clinic in April 2018 because her RLS symptoms worsened for 2 months such that she couldn't sleep at all. She was diagnosed with RLS according to the IRLSSG criteria [7] and her IRLSRS score was 32. She was prescribed pramipexole 0.125 mg/d for therapy. Two weeks later, she experienced stereotyped behaviors such as repetitively tidying up the table. She also showed symptoms of compulsively searching for food at home and picking up various trash at home. Her husband described that she also presented excessive sexual behaviors, such as asking him to fondle her pudendum, which troubled her and her family. Her symptoms gradually were relieved after the withdrawal of pramipexole. On subsequent telephone follow-ups, the patient never presented impulsive behaviors any more.

## 3 Discussion

### 3.1 ICDs and ICD-related disorders

Case 1 showed a single ICD symptom of hypersexuality. Case 2 presented more addictive behaviors, including punding, binge eating, hoarding, and compulsive sexual disorder. ICDs may have an overlap with obsessive-compulsive disorder (OCD) and other impulsive and compulsive behaviors [8]. Previous studies reported that nearly half of RLS patients with ICDs may present at least one addictive behavior [5,6]. Features of ICD subtypes and underlying cross-over mechanisms with OCD and other compulsive behaviors need further investigation.

### 3.2 Dopaminergic agents associated with ICDs

The central mesolimbic pathway is considered to be involved in PD patients with ICDs, although the exact pathophysiology of ICDs has not been elucidated yet [1]. The prevalence rates of ICDs range from 7% to 20% in RLS patients with dopaminergic therapy [[4], [5], [6]]. The two cases we report here are both dopamine agonist (pramipexole) treatment-induced ICDs that were relieved after discontinuation of the dopaminergic agents. In line with previous studies, ICDs usually resolve after reducing the dosage or completely discontinuing dopaminergic medications [9]. Notably, in the first case, after withdrawal of pramipexole, levodopa therapy alone also caused hypersexuality. Much evidence shows non-ergot dopamine agonists such as pramipexole and ropinirole are the most common causes of ICDs, while levodopa causes ICDs to a lesser extent than dopamine agonist treatment [4]. This may be partly because pramipexole and ropinirole have relatively higher selectivity and affinity for dopamine D3 receptors, which are extensively expressed in the dopaminergic mesolimbic system and associated with worse ICDs [10].

It is noteworthy that the two RLS patients developed ICD symptoms even though they received relatively lower dosages of pramipexole (0.25 mg per day in case 1 and 0.125 mg per day in case 2). This is consistent with previous studies that also observed low doses of dopamine agonists may induce ICDs in RLS [5,11], although an earlier study recorded a dosage-related effect of pramipexole on ICDs in RLS [4].

### 3.3 Age as a factor for ICDs in patients with RLS

It is reported that younger age and earlier disease onset are correlated with ICDs in PD, although the mechanisms are unclear [12]. Age as a risk factor for ICDs in RLS has not been reported yet. Coincidentally, the two RLS cases we describe here both developed ICDs after age 80. With the aging of society, elderly populations may constitute a small subgroup of RLS patients, including RLS patients who have had the disease for decades and are now over 80 years old (such as case 2), and those who develop RLS in older age (such as case 1). Elderly patients present with declined physiological compensatory mechanisms. They show reduced creatinine clearance and other special pharmacokinetic characteristics that may cause them to reach a therapeutic drug level at lower dosage [13], although no consistent dose effect has been described for ICDs [1,5].

Dopamine agonists are first-line agents for RLS treatment; however, the guidelines for dopamine agonists and other dopaminergic drugs are limited because most randomized controlled trials exclude very elderly RLS patients [3]. Caution should be taken in clinical practice when prescribing these dopaminergic medications in very elderly patients.

In summary, this study broadened our understanding of ICDs in RLS in very elderly patients. Dopamine agonists and other dopaminergic pharmacotherapies should be used cautiously in elderly RLS patients with ICDs. Because ICDs worsen quality of life and function, clinicians should screen carefully for ICDs in elderly RLS patients. Prompt identification of ICDs and discontinuation of dopaminergic agents are extremely imperative in ICD sufferers.

## Declaration of competing interest

Nothing to declare.
